# Supplementary material for: A signal peptide peptidase is required for ER-symbiosome proximal association and protein secretion
Source: Nat Commun. 2023 Jul 19;14:4355. doi: 10.1038/s41467-023-40008-3 (PMC10356799; doi:10.1038/s41467-023-40008-3)
Supplement: Supplementary file 1 — Supplementary Information [file 41467_2023_40008_MOESM1_ESM.pdf]

## Supplementary information

A signal peptide peptidase is required for ER-symbiosome proximal association and protein secretion

**Authors:**

Jian Yang[1], Niu Zhai[2], Yuhui Chen[3], Luying Wang[1], Rujin Chen[3], Huairong Pan[1*]

**Contacting information:**

[1]College of Biology, Hunan University, Changsha, 410082, China

[2] Zhengzhou Tobacco Research Institute of CNTC, Zhengzhou, 450001, China

[3]College of Life Sciences, Lanzhou University, Lanzhou, 730000, China

**Author for correspondence:** Huairong Pan, email: hrpan@hnu.edu.cn.

## Supplemental Figures

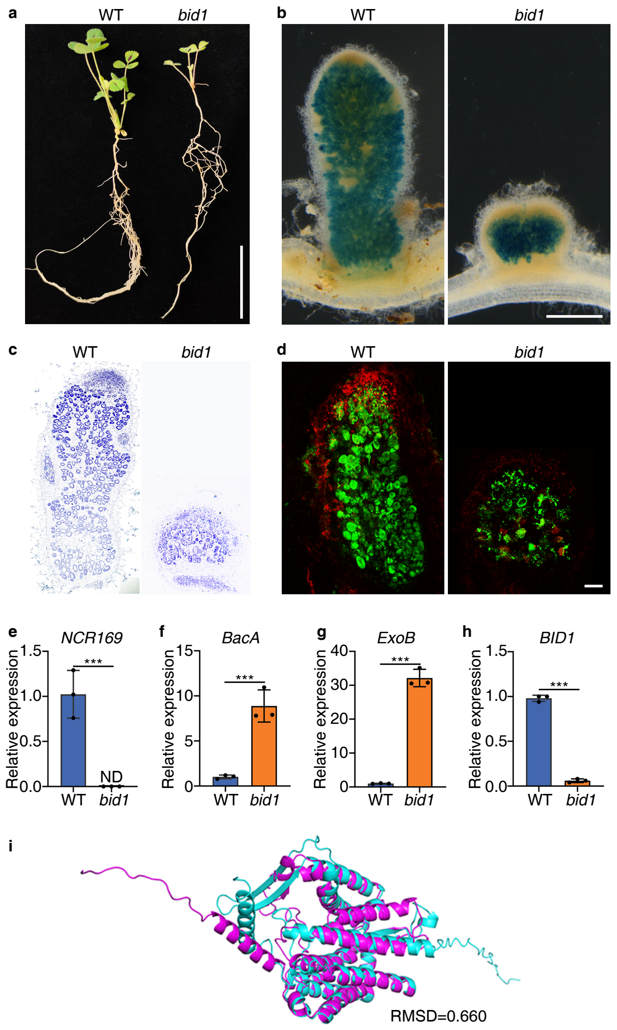


Supplemental Figure 1. *bid1* plants, nodules, nodule cells and symbiosomes are smaller comparing with WT.

a, *bid1* plants were smaller compared with WT when inoculated with rhizobia. WT and *bid1* plants were grown in green zeolite under nitrogen nutrient free condition, and were inoculated with *S. meliloti* ABS7 *hemA::LacZ* and plant growth were determined at 21 dpi. Representative plants of both genotypes were shown. Bar= 2 cm. b, Symbiosomes were still viable in *bid1* nodules. Nodules from a were hand sectioned and stained with X-gal. Bar=1 mm. c, Overall structural comparison of WT and *bid1* nodules. Nodule zones were altered in *bid1* and nodule cells were smaller. WT and *bid1* plants were inoculated with ABS7 *hemA::LacZ*. Nodules at 21 dpi were sectioned into 5 μm slides and stained with toluidine blue. Bar=300 μm. d, Symbiosomes in *bid1* nodule cells were not differentiated. Nodules from a were stained with SYTO9 (to stain live symbiosome) and PI (to stain dead symbiosome). Bar=100 μm. e to g, Expression levels of rhizobium *ExoB* and *BacA*, and Medicago *NCR169* in WT and *bid1* nodule cells. RNA was extracted from WT and *bid1* nodules inoculated with ABS7 *hemA::LacZ* for 21 days. h, The expression level of *MtrunA17_Chr1g0147151* was much lower in *bid1* compared with WT in a qRT-PCR assay. The same samples from e were used. ND, not detected. For e to h, data were

represented as means ± SEM of three independent amplifications, “***”, $P<0.001$ in Student’s t-test. i, The predicted structures of BID1 and human SPP protein are very similar. Protein structures of BID1 and human SPP protein were predicted using Alphafold Protein Structure Database (https://www.alphafold.ebi.ac.uk/) and merged together with PyMol (Version 2.5.3). BID1 and human SPP structures are labeled in yellow and cyan respectively. RMSD, root mean square deviation. For a-h, experiments were repeated 3 times with similar results.

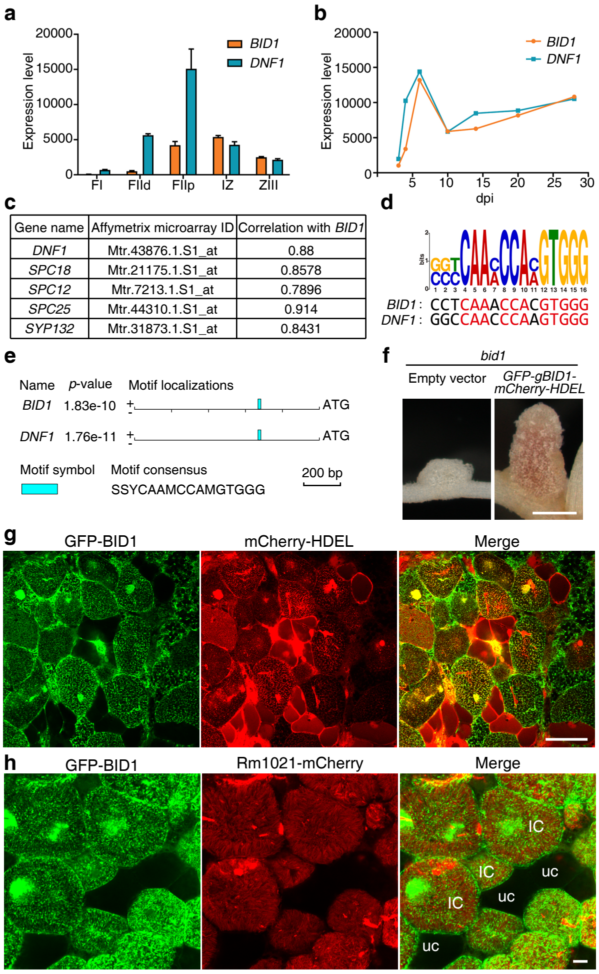


Supplemental Figure 2. *BID1* co-expresses with components of *DNF1* nodule-specific SPC, and is specific to infected cells.

a, Expression levels of *BID1* and *DNF1* in different zones of Medicago nodules. Raw data was acquired from the Symbimics website. The database designates and names nodule zones differently comparing to Fig. 2d. FI, nodule meristem, FIId, infection zone, FIIp, differentiation zone, IZ (interzone), the region between differentiation zone and fixation zone, ZIII, fixation zone. Y-axis indicates normalized gene expression level based on RNA-Seq data. b, Expression levels of *BID1* and *DNF1* in nodules at different time points post inoculation. Gene expression data was obtained from Medicago Gene Expression Atlas. Y-axis, normalized gene expression levels acquired from Microarray data. c, Correlation of gene expression between *BID1* with components of *DNF1* nodule specific SPC and *SYP132* isoforms. Correlation values were obtained from Medicago Gene Expression Atlas. Please note that one single probe represents *SYP132A* and *SYP132C*, two isoforms of *SYP132* together. d, *BID1* and *DNF1* contain common cis elements in their promoter regions. Conserved nucleotides in promoter sequences of *BID1* and *DNF1* were analyzed using motif discovery tools in MEME database (https://meme-suite.org/meme/tools/meme). The conserved nucleotides were shown in full scale letters. e, Positions of identified cis element in d in promoter regions of *BID1* and *DNF1*. Positions of the conserved cis element were shown by cyan boxes. Numbers indicate distances to start codons. f, *pBID1::GFP-gBID1-UBQ10::mCherry-HDEL-pKGW*

construct could complement *bid1* fix- phenotype. *bid1* and transgenic plants were inoculated with ABS7 *hemA::LacZ* for 21 days, representative nodules were imaged. Bar=750 μm. g, GFP-BID1 was expressed in large cells only. Nodules from f were analyzed by confocal microscopy. Bar=50 μm. h, Confocal images showing the expression of GFP-BID1 protein driven by its native promoter. *bid1* plants were transformed with *pBID1::GFP-gBID1* and inoculated with *S. meliloti* Rm1021-mCherry. Nodules at 21 dpi were analyzed through confocal microscopy. GFP fluorescence could only be detected in large cells with mCherry fluorescence. Bar=10 μm. IC, infected cells, UC, uninfected cells. Experiments were repeated more than three times with similar results.

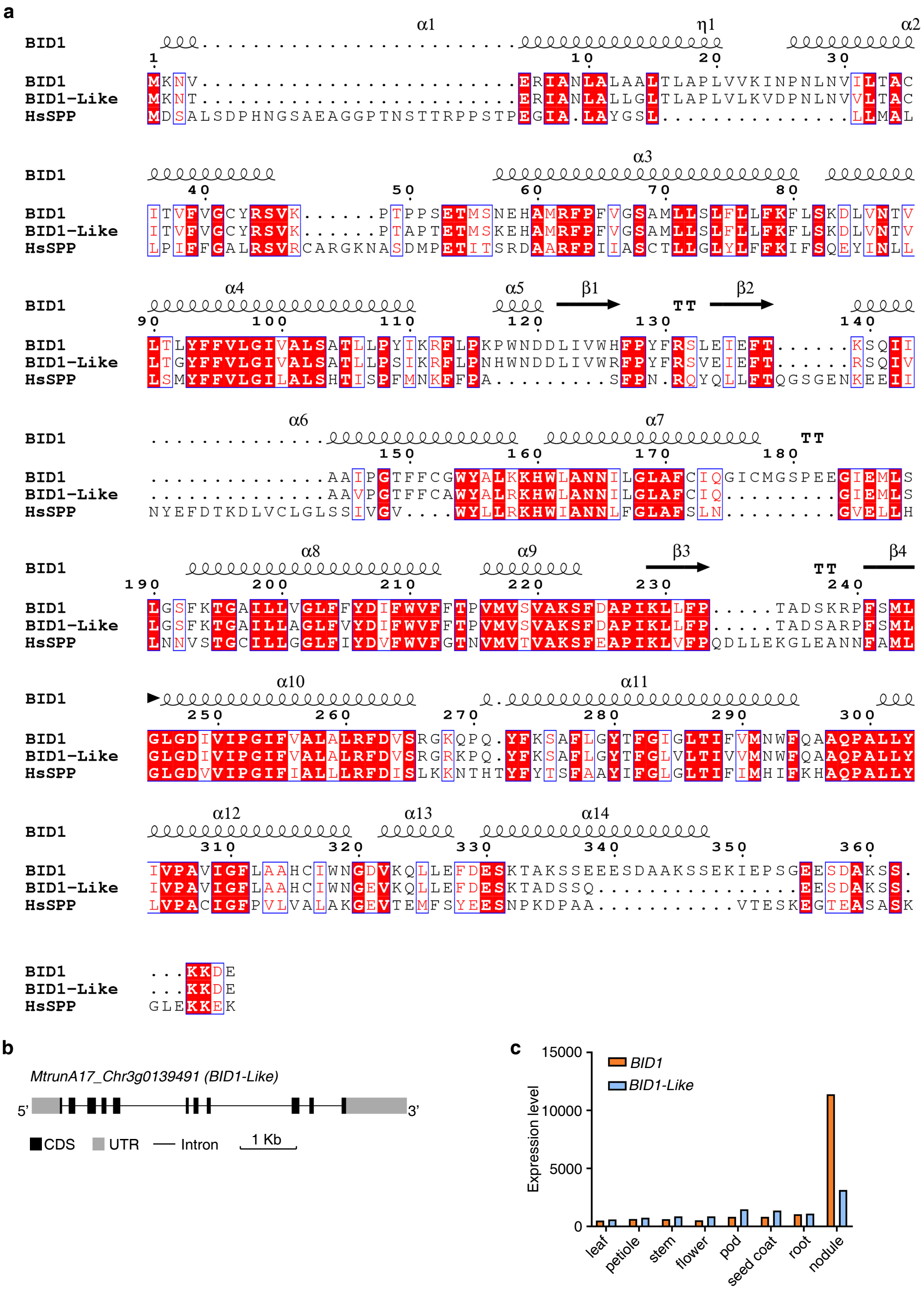


Supplemental Figure 3. In Medicago *BID1L* is the housekeeping homolog of *BID1*.

a, *BID1* and *BID1L* showed very high sequence similarity to human SPP protein in the alignment assay. The protein sequences were aligned through MAFFT (https://mafft.cbrc.jp/alignment/software/) and visualized via ESPript. Red background indicated identical residues, and similar residues were labelled in red. Conserved regions were labelled by blue boxes. Secondary structures in BID1 protein were labelled on top of aligned sequences. b, the gene structure of *BID1L*. c, In Medicago *BID1L* expresses stably in various tissues. Gene expression data of *BID1* and *BID1L* was acquired from Medicago gene expression atlas (https://mtgea.noble.org/v3/). Y-axis, normalized gene expression levels basing on Microarray data.

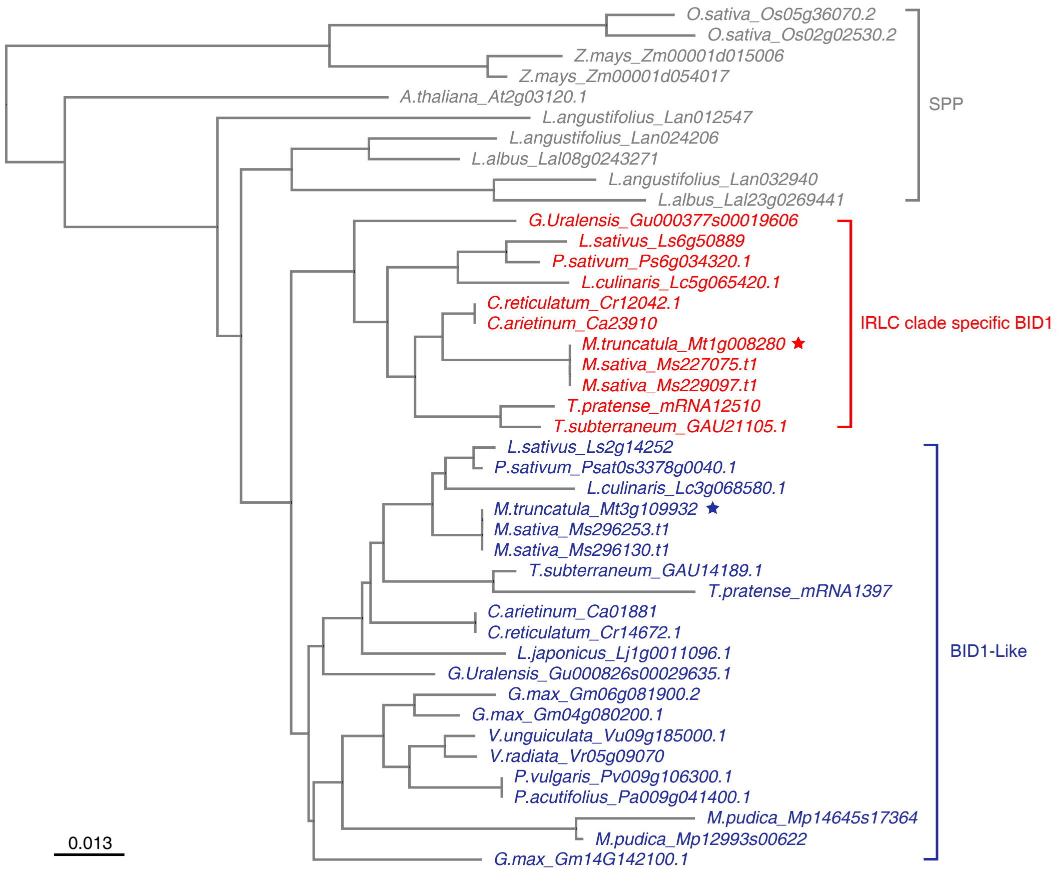


Supplemental Figure 4. *BID1* is specific to IRLC-clade legume species.

Orthologues of *BID1* could only be found in IRLC-clade legumes, while *BID1L* orthologues could be found in monocots and dicots, including legumes forming determinate and indeterminate nodules. Some species, e.g., *G. max*, even contains two *BID1L* copies. Phylogenic tree was made using the neighbor-joining method in MEGA 11 (https://megasoftware.net/). The bootstrap values (percent as numbers) were determined basing on results from 1000 replicates. Scale bar, 0.1 nucleotide substitutions per sequence position. Accession numbers of genes used were listed in Supplemental Table 3.

**a**

| *Arabidopsis thaliana* | *Medicago truncatula* | Gene name |
|---|---|---|
| *At3g10800* | *Medtr7g088890* | *bZIP28a* |
| | *Medtr7g116890* | *bZIP28b* |
| *At5g62390* | *Medtr4g036110* | *BAG7a* |
| | *Medtr7g012940* | *BAG7b* |
| | *Medtr8g028265* | *BAG7c* |
| *At1g42900* | *Medtr7g088090* | *bZIP60a* |
| | *Medtr8g086020* | *bZIP60b* |
| *At1g09080* | *Medtr8g099795* | *BIP3a* |
| | *Medtr4g130540* | *BIP3b* |
| *At1g72280* | *Medtr2g048900* | *ERO1* |
| *At5g24360* | *Medtr5g024510* | *IRE1* |

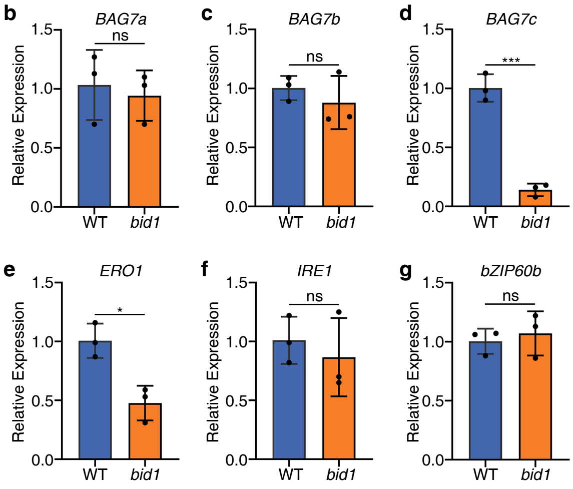


Supplemental Figure 5. Expression of ER stress marker genes in WT and *bid1* nodule cells.

a, A table showing Medicago orthologues of reported Arabidopsis ER stress response genes. When there were more than one orthologues, the genes were named in alphabetical order. b to f, qRT-PCR assay results of *BAG7a*, *BAG7b*, *BAG7c*, *ERO1*, *IRE1*, *bZIP60b* in WT and *bid1* nodule cells. *BAG7a*, *BAG7b* and *BAG7c* are Arabidopsis BAG orthologues. Nodules inoculated with ABS7 *hemA::LacZ* for 14 days were used for the experiment. "***", P<0.001 in Student's t-test, "ns", not significant. For b to f, data were presented as means ± SEM of three independent amplifications. Note that expression levels of some ER stress response genes did not change in *bid1* nodules. Experiments were repeated 5 times with similar results.

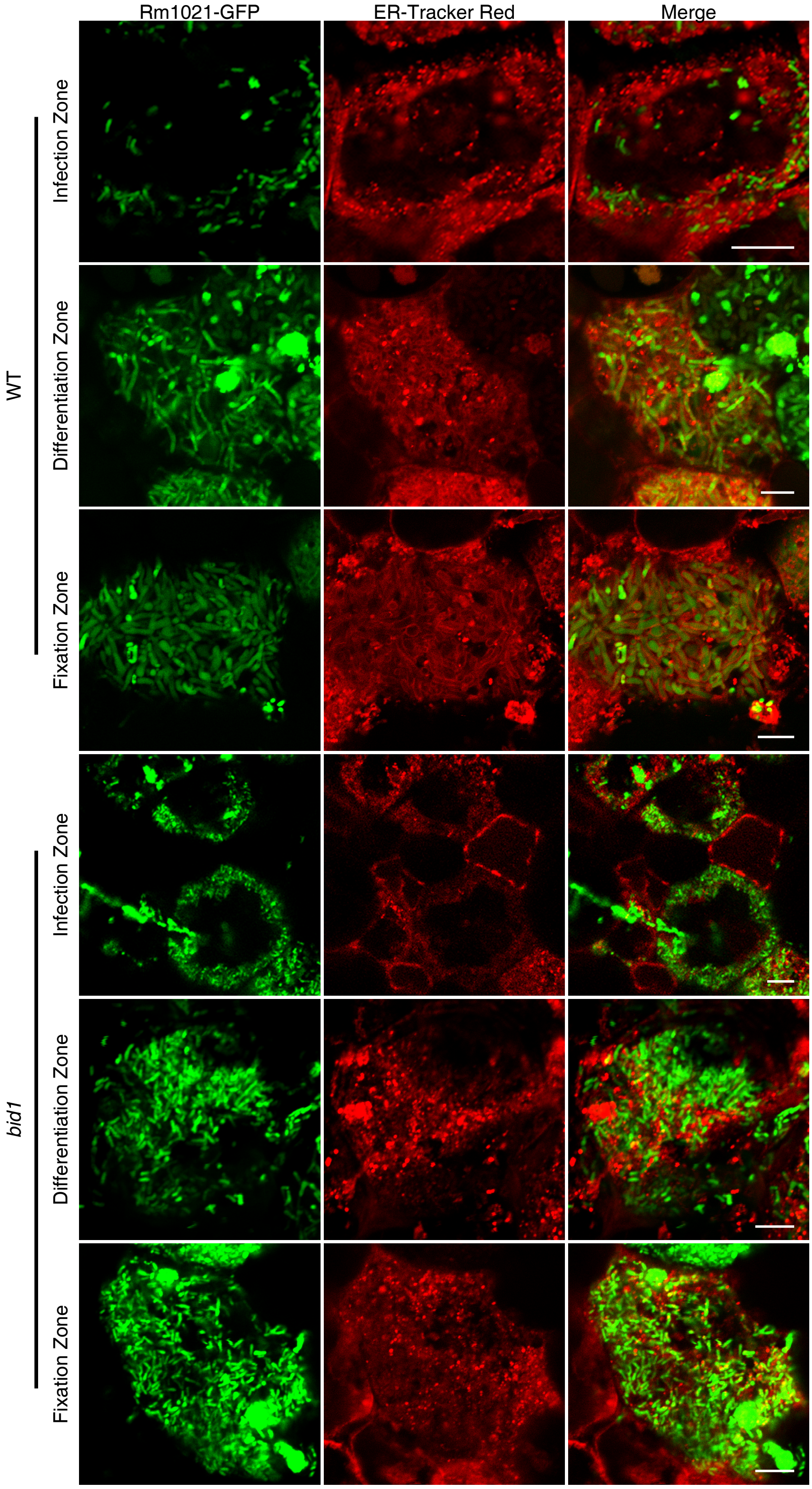


Supplemental Figure 6. ER-Tracker Red staining assays demonstrated structural differences between ER and symbiosomes in WT and *bid1* nodule cells.

Confocal microscopy assay results of infection, differentiation and fixation zone nodule cells of WT and *bid1* respectively. Green fluorescence showed GFP-labelled symbiosomes, and red was ER-Tracker Red stained ER structure. Bar= 10 μm. 14 dpi nodules inoculated with *S. meliloti* Rm1021 *pHC60-GFP* were used for the experiment. Experiments were repeated 3 times with identical results.

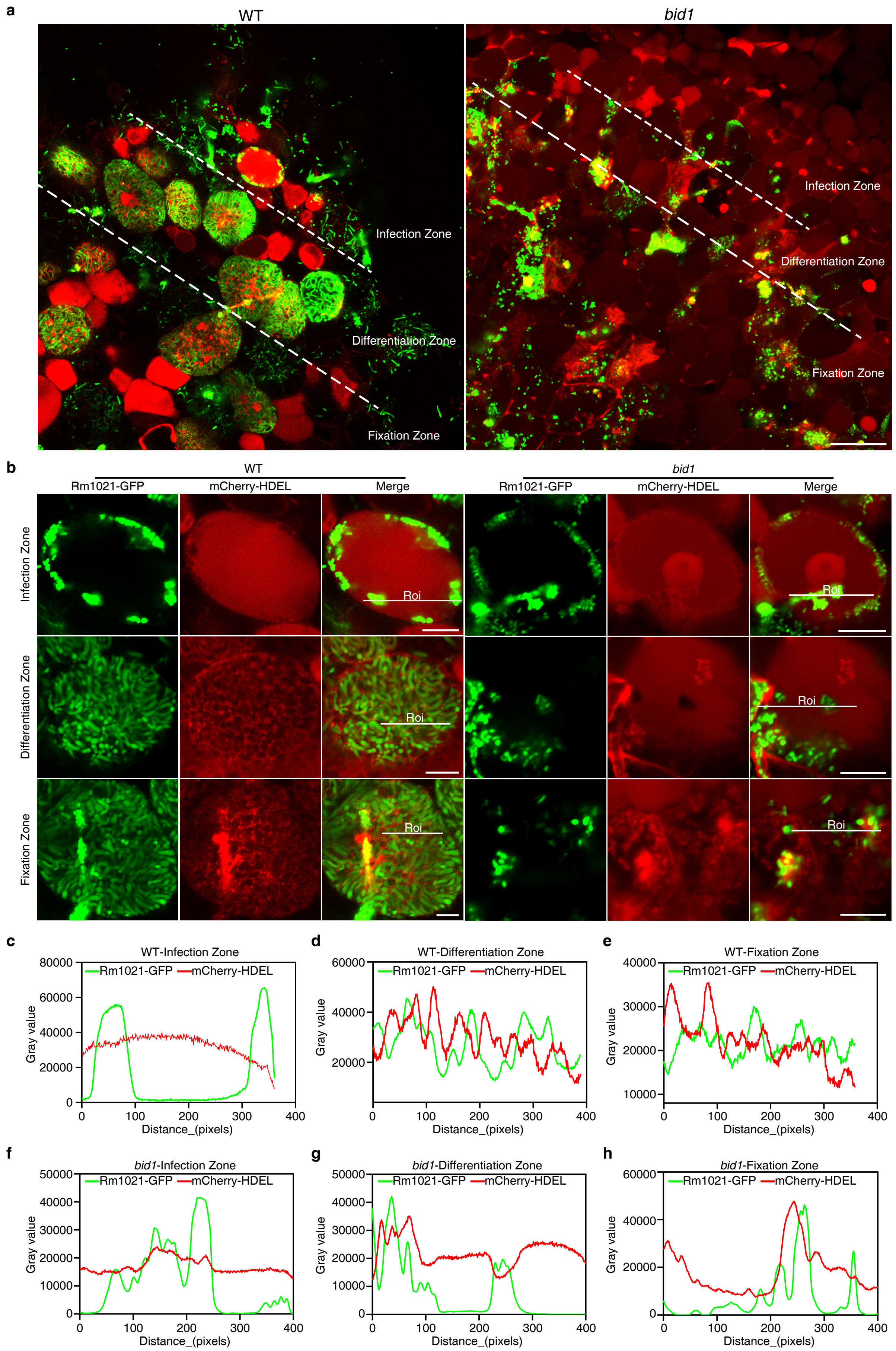


Supplemental Figure 7. Zone-by-zone comparison of structural relationships between ER and

symbiosomes in WT and *bid1* nodule cells.

a, Overall structural differences between WT and *bid1* nodules in ER and symbiosome structures. Green, GFP-labelled symbiosome, red, mCherry-HDEL lebelled ER. Dashed white lines divided nodules into 3 major zones, Infection Zone, Differentiation Zone and Fixation Zone. Bar= 50 μm. b, ER-symbiosome proximal association could be detected in WT differentiation and fixation zone nodule cells, but not *bid1*. Shown are confocal microscopy assay pictures of WT and *bid1* infection, differentiation and fixation zone cells. Bar=10 μm. Roi, Region of interest. c to h, Fluorescent intensity assay results showing the spacial relationship between ER and symbiosomes in different zones of WT and *bid1* nodules. In WT differentiation and fixation zones, symbiosome GFP fluorescence peaked closely to mCherry-HDEL, but not in WT infection zone and *bid1* nodule cells. Fluorescent intensity in white boxes indicated regions in b was measured as grey value of pixels using ImageJ. Similar results were shown from experiments repeated at least 3 times.

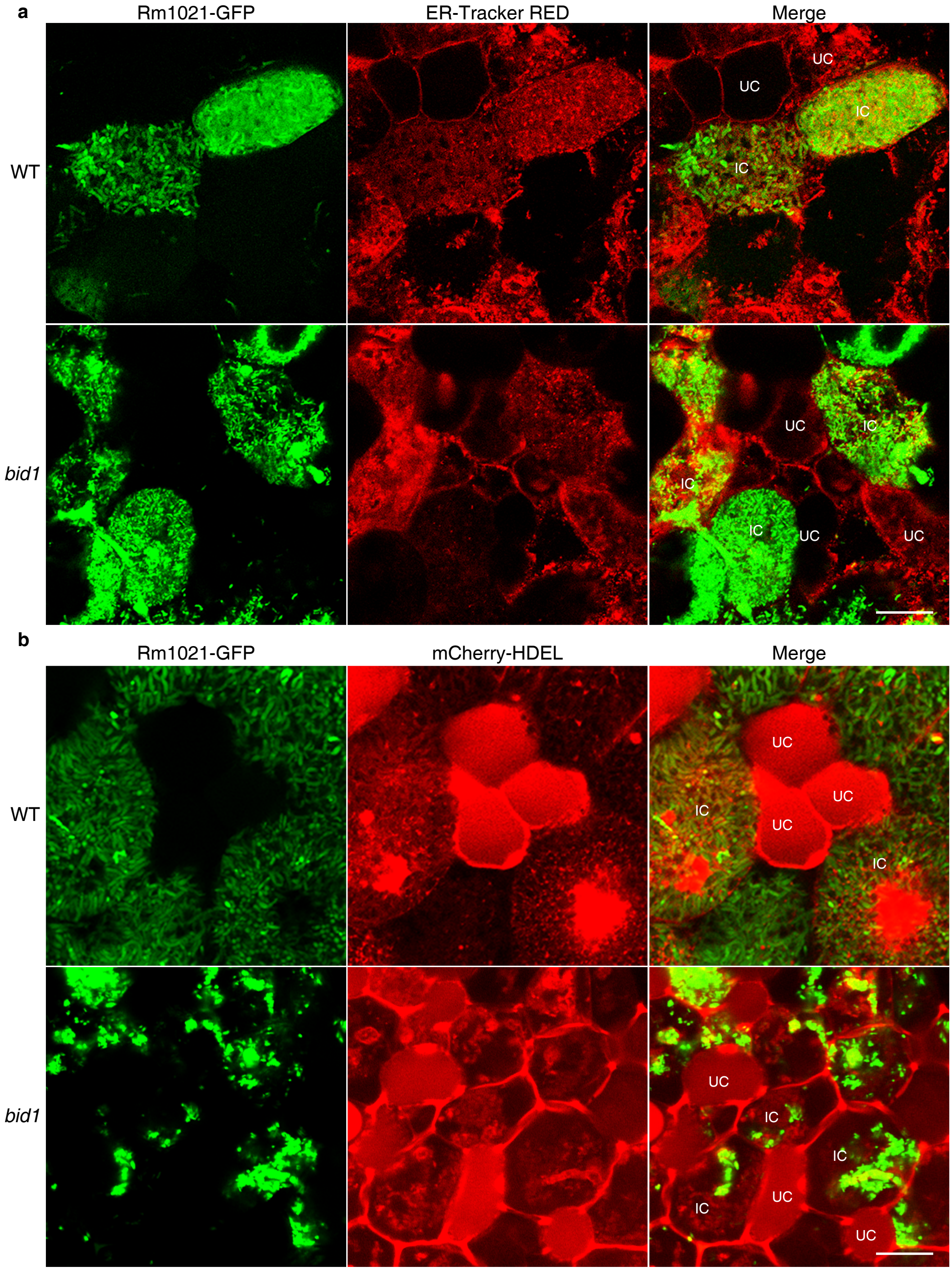


Supplemental Figure 8. ER structural reconfiguration is specific to infected nodule cells.

a and b, Comparison of ER structures of infected and uninfected cells in WT and *bid1* fixation zones respectively. 14 dpi nodules inoculated with Rm1021 *pHC60-GFP* were used for the experiment. Nodules were sectioned, stained and observed under confocal microscope. For a, nodule cells were stained with ER-Tracker Red. For b, plants transformed with mCherry-HDEL marker were used. Bar=50 μm. IC, infected cells, UC, uninfected cells. Similar results were obtained from experiments repeated 3 times.

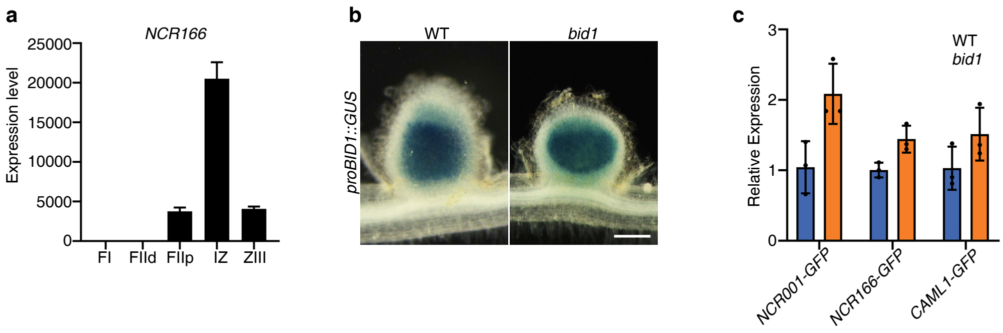


Supplemental Figure 9. The *BID1* promoter can be used to drive the expression of marker proteins in *bid1*.

a, Expression pattern of *NCR166* in nodule zones. Normalized expression data basing on RNA-Seq was acquired from Symbimics website. b, GUS reporter assay to demonstrate the full activity of *BID1* promoter in *bid1* nodules. *pBID1::GUS* reporter expressing WT and *bid1* plants were inoculated with ABS7 *hemA::LacZ*, GUS staining was performed at 14 dpi. Bar=500 μm. c, When expressed under *BID1* promoter, the expression levels of *NCR001-GFP*, *NCR166-GFP* and *CAML1-GFP* were even slightly higher in *bid1* compared with WT. 14 dpi *pBID1::NCR001-GFP*, *pBID1::NCR166-GFP* and *pBID1::CAML1-GFP* expressing WT and *bid1* nodules inoculated with ABS7 *hemA::LacZ* were used for qRT-PCR assay. Data were represented as means ± SEM of three independent amplifications. Experiments were repeated three times with similar results.

Supplemental Table 1. Plasmids used in this study

| Construct | Description | Source |
| --- | --- | --- |
| *pBID1-pMDC163* | promoter-GUS | This study |
| *pBID1-GFP-gBID1-pKGW* | complementation | This study |
| *pBID1-gBID1-GFP-pKGW* | complementation | This study |
| *35S-mCherry-HDEL* | endoplasmic reticulum marker | Prof. Lijing Liu, Shandong University |
| *pUBQ10-mCherry-HDEL-pKGW* | endoplasmic reticulum marker | This study |
| *pBID1-GFP-gBID1-UBQ10-mCherry-HDEL-pKGW* | subcellular localization | This study |
| *35S-mCherry-HDEL-pMDC32* | subcellular localization | This study |
| *35S-GFP-BID1-pMDC32* | subcellular localization | This study |
| *pBID1-NCR001-GFP-PKGW* | marker gene subcellular localization | This study |
| *pBID1-NCR166-GFP-PKGW* | marker gene subcellular localization | This study |
| *pBID1-CAML1-GFP-PKGW* | marker gene subcellular localization | This study |

Supplemental Table 2. Primers used in this study

| **Construct** | **Primer name** | **Primer sequence(5'-3')** |
|---|---|---|
| *pBID1-pMDC163* | pBID1-pMDC163-F | agtgccaagctctagttatgcctctagtttgtcctgtacc |
| | pBID1-pMDC163-R | cccggggatcgatcctctagacttgattcaccctgcaatcacaa |
| *pBID-gBID1-GFP-pKGW* | BID1-pMDC107-F | gcaggtcgactctagtgcctctagtttgtcctgtacc |
| | BID1-pMDC107-R | ccccctcgaggcgcgttcatctttttgctagatttggcatc |
| | BID1-GFP-PKGW-F | agcaccaaccacaacgacgtctgcctctagtttgtcctgtacctg |
| | BID1-GFP-PKGW-R | aagcttagcttgagcttggattcatctttttgctagatttggcatc |
| *pBID-GFP-gBID1-pKGW* | GFP-puc18-F | aggtcgactctagaggatccatgggcaaaggagaagaacttttc |
| | GFP-puc18-R | acgaattcgagctcggtacctttgtatagttcatccatgccatgt |
| | gBID1-puc18-GFP-F | atgaactatacaaaggtaccatgaaaaacgttgagaggattgcg |
| | gBID1-puc18-GFP-R | atgaccatgattacgaattcacatgcatgatccaacaatcccctc |
| | pBID1-puc18-gBID1-F | caagcttgcatgcctgcaggtgcctctagtttgtcctgtacc |
| | pBID1-puc18-gBID1-R | tctcctttgcccatggatcccttgattcaccctgcaatcacaa |
| | pBID1-GFP-gBID1-PKGW-F | tagatgcatgctcgagtgcctctagtttgtcctgtacc |
| | pBID1-GFP-gBID1-PKGW-R | ctcaagctaagcttgaattctcttaaaaatcaaaatgttatcatgttaat |
| *pUBQ10-mCherry-HDEL-PKGW* | mCherry-HDEL-puc18-F | gcctgcaggtcgactctagaatggtgagcaagggcgagg |
| | mCherry-HDEL-puc18-R | atgaccatgattacgaattcgatctagtaacatagatgacaccgc |
| | pUBQ10-puc18-mCherry-HDEL-F | aggttgccggatcctctagactgttaatcagaaaaactcag |
| | pUBQ10-puc18-mCherry-HDEL-R | cccttgctcaccattctagactgttaatcagaaaaactcag |
| | pUBQ10-mCherry-HDEL-PKGW-F | tccaagctcaagctaagcttgttgtggttggtgctttcct |
| | pUBQ10-mCherry-HDEL-PKGW-R | tctagatgcatgctcgaggatctagtaacatagatgacaccgc |
| *pBID1-GFP-gBID1-UBQ10-mCherry-HDEL-pKGW* | pUBQ10-mCherry-HDEL-pBID1-GFP-gBID1-PKGW-F | acaaactagaggcagacgtcgttgtggttggtgctttccttac |
| | pUBQ10-mCherry-HDEL-pBID1-GFP-gBID1-PKGW-R | tctagatgcatgctcgaggatctagtaacatagatgacaccgc |
| *35S-GFP-BID1-pMDC32* | 35s-GFP-BID1-pMDC32-F | ccgacggccagtgccaagcttggcaattgagacttttcaacaaagg |
| | 35s-GFP-BID1-pMDC32-R | gcggccgctctagaactagtacatgcatgatccaacaatcccctc |

| **Construct** | **Primer name** | **Primer sequence(5'-3')** |
|---|---|---|
| *pBID1-NCR001-GFP-PKGW*<br>*pBID1-NCR166-GFP-PKGW*<br>*pBID1-CAML1-GFP-PKGW* | GFP-PKGW-F | tagatgcatgctcgaggaattcgtcgacat gggcaaaggagaagaactt |
| | GFP-PKGW-R | agctcaagctaagcttttatttgtatagttcat ccatgccatg |
| | pBID1-GFP-pKGW-F | tatgataattcgagggtacctgcctctagttt gtcctgtacc |
| | pBID1-GFP-pKGW-R | tcctcgagcatgcatctagacttgattcacc ctgcaatcacaa |
| | pBID1-CAML1-GFP-PKGW-F | cagggtgaatcaagtctagaatgggttact caaattcatatttgt |
| | pBID1-CAML1-GFP-PKGW-R | ttgcccatgtcgacgaattcgaccacaatg gatccacc |
| | pBID1-NCR001-GFP-PKGW-F | cagggtgaatcaagtctagaatggctcaa tttcttctgtttg |
| | pBID1-NCR001-GFP-PKGW-R | ttgcccatgtcgacgaattctggcccttcat atgaaccttc |
| | pBID1-NCR166-GFP-PKGW-F | cagggtgaatcaagtctagaatggctaaa attatcaattttgtttataatatg |
| | pBID1-NCR166-GFP-PKGW-R | ttgcccatgtcgacgaattctgtgagcaca cgatttgtttc |

| RT primer name | Primer sequence(5'-3') |
|---|---|
| PDF2-RT-F | gggagcaagatttggttgagc |
| PDF2-RT-R | ctccggtgcactagggtagg |
| 16s-RT-F | gataagccgagaggaaggtg |
| 16s-RT-R | gtgtagcccagcccgtaag |
| NCR001-RT-F | gaagcggcgtttgagaggac |
| NCR001-RT-R | tcaccttcggacagttagcgt |
| NCR169-RT-F | cacctgtttcttgttgtaatcttcg |
| NCR169-RT-R | acacaaacattttcaacgcatttcc |
| ExoY-RT-F | gacccgcaagcttcagaacga |
| ExoY-RT-R | gacgctcatttcaccgcgaat |
| ExoB-RT-F | ttcagcttgcggcaaagggttac |
| ExoB-RT-R | atcatcgccgcgaagtgc |
| CtrA-RT-F | tcggggaagaaggcgtcgat |
| CtrA-RT-R | tagtcgtcggcgccgaagcc |
| BacA-RT-F | gtcggtgtgctcaacctctt |
| BacA-RT-R | ccgggagaaaggcgatcagc |
| BID1-RT-F | cgttgagaggattgcgaatttgg |
| BID1-RT-R | aaacacagtgatgcaagcagtc |
| bZIP28a-RT-F | atgcaacagtggttccgtg |
| bZIP28a-RT-R | ggaaaacttcagtacacatgccag |
| bZIP28b-RT-F | gtttatactcaccctgcagttcc |
| bZIP28b-RT-R | ctccaggctcagcactctca |
| BAG7a-RT-F | ttgtcttgctcctcaccgttg |
| BAG7a-RT-R | cttccagctcatccaccattg |
| BAG7b-RT-F | aacccatttccgttctttcctc |
| BAG7b-RT-R | cgaccttttcccatccacaatc |
| BAG7c-RT-F | agctgtttatcgcggttatctag |
| BAG7c-RT-R | cctgcaaagtcacaagctctc |

| RT primer name | Primer sequence(5'-3') |
|---|---|
| bZIP60a-RT-F | ctgctatgctgagaaccatgc |
| bZIP60a-RT-R | gcgacagtggaaggggtaga |
| bZIP60b-RT-F | gtggagaggaggcagaagagg |
| bZIP60b-RT-R | gggagctgagctagttctccg |
| bZIP60c-RT-F | tggcacaagctctcttcaaaactct |
| bZIP60c-RT-R | atcttcgtgctgattctctgttgg |
| IRE1-RT-F | attgaatgtggtgatgactggg |
| IRE1-RT-R | ggcgtaacagcaacataatcagc |
| BIP3a-RT-F | gatggctggctcgtggaaac |
| BIP3a-RT-R | ggttgcttcctcttttgcattgg |
| BIP3b-RT-F | cgaaatcatccctaacgaccaag |
| BIP3b-RT-R | cgcttagcatcgaagacggt |

Supplemental Table 3. Gene locus list

| Gene name | Gene locus |
|---|---|
| *16S* | *Smc02676* |
| *ExoY* | *Smb20946* |
| *ExoB* | *Smb20942* |
| *CtrA* | *Smc00654* |
| *BacA* | *Smb20999* |
| *PDF2* | *Medtr6g084690* |
| *NCR001* | *Medtr6g463200* |
| *NCR169* | *Medtr7g029760* |
| *CAML1* | *Medtr3g055570* |

| Phylogenetic tree of BID1 | |
|---|---|
| Species | Transcript name |
| *Arabidopsis thaliana* | *A.thaliana_At2G03120.1* |
| *Cicer arietinum* | *C.arietinum_Ca01881* |
| *Cicer arietinum* | *C.arietinum_Ca23910* |
| *Cicer reticulatum* | *C.reticulatum_Cr12042.1* |
| *Cicer reticulatum* | *C.arietinum_Ca23910* |
| *Glycine max* | *G.max_Gm04G080200.1* |
| *Glycine max* | *G.max_Gm06G081900.2* |
| *Glycine max* | *G.max_Gm14G142100.1* |
| *Glycyrrhiza Uralensis* | *G.Uralensis_Gu000377s00019606* |
| *Glycyrrhiza Uralensis* | *G.Uralensis_Gu000826s00029635.1* |
| *Lathyrus sativus* | *L.sativus_Ls6g50889* |
| *Lathyrus sativus* | *L.sativus_Ls2g14252* |
| *Lupinus albus* | *L.albus_Lal08g0243271* |
| *Lupinus albus* | *L.albus_Lal23g0269441* |
| *Lupinus angustifolius* | *L.angustifolius_Lan012547* |
| *Lupinus angustifolius* | *L.angustifolius_Lan024206* |
| *Lupinus angustifolius* | *L.angustifolius_Lan032940* |
| *Lens culinaris* | *L.culinaris_Lc5g065420.1* |
| *Lens culinaris* | *L.culinaris_Lc3g068580.1* |
| *Lotus japonicus* | *L.japonicus_Lj1g0011096.1* |
| *Medicago Sativa* | *M.sativa_Ms227075.t1* |
| *Medicago Sativa* | *M.sativa_Ms229097.t1* |
| *Medicago Sativa* | *M.sativa_Ms296130.t1* |
| *Medicago Sativa* | *M.sativa_Ms296253.t1* |
| *Medicago Truncatula* | *M.truncatula_Mt1g008280* |
| *Medicago Truncatula* | *M.truncatula_Mt3g109932* |
| *Mimosa Pudica* | *M.pudica_Mp14645S17364* |
| *Mimosa Pudica* | *M.pudica_Mp12993S00622* |
| *Oryza sativa* | *O.sativa_Os02g02530.2* |
| *Oryza sativa* | *O.sativa_Os05g36070.2* |
| *Phaseolus acutifolius* | *P.acutifolius_Pa009G041400.1* |
| *Pisum sativum* | *P.sativum_Psat0s3378g0040.1* |
| *Pisum sativum* | *P.sativum_Ps6g034320.1* |
| *Phaseolus vulgaris* | *P.vulgarisv_Pv009G106300.1* |
| *Trifolium pratense* | *T.pratense_mRNA12510* |
| *Trifolium pratense* | *T.pratense_mRNA1397* |
| *Trifolium subterraneum* | *T.subterraneum_GAU14189.1* |

| Species | Transcript name |
|---|---|
| *Trifolium subterraneum* | *T.subterraneum_GAU21105.1* |
| *Vigna radiata* | *V.radiata_Vr05g09070* |
| *Vigna unguiculata* | *V.unguiculata_Vu09G185000.1* |
| *Zea mays* | *Z.mays_Zm00001d015006* |
| *Zea mays* | *Z.mays_Zm00001d054017* |
