# Supplementary material for: A signal peptide peptidase is required for ER-symbiosome proximal association and protein secretion
Source: Nat Commun. 2023 Jul 19;14:4355. doi: 10.1038/s41467-023-40008-3 (PMC10356799; doi:10.1038/s41467-023-40008-3)
Supplement: Supplementary file 3 — Description of Additional Supplementary Files [file 41467_2023_40008_MOESM3_ESM.pdf]

Description of Additional Supplementary Files

Supplementary Movie 1 and 2. Videos to show ER and symbiosome structures in WT and *bid1* fixation zone nodule cells respectively. The videos were generated through serial sectioning. 69 sections were used for WT, 64 were used for *bid1*. Pictures of serial sections were aligned by ImageJ software. 14 dpi nodules inoculated with ABS7 *hemA::LacZ* were used for observation.

Supplemental Movie 3 and 4. Videos demonstrating 3D structural modelling of ER and symbiosome in WT and *bid1* fixation zone cells respectively. Symbiosomes were labelled in blue and ER in orange red. Hand labelling of ER and symbiosome structures in serial section pictures and subsequent 3D model construction were done by Imaris software.
