# Supplementary material for: A signal peptide peptidase is required for ER-symbiosome proximal association and protein secretion
Source: Nat Commun. 2023 Jul 19;14:4355. doi: 10.1038/s41467-023-40008-3 (PMC10356799; doi:10.1038/s41467-023-40008-3)
Supplement: Supplementary file 8 — Reporting Summary [file 41467_2023_40008_MOESM8_ESM.pdf]

nature portfolio

Corresponding author(s): Huairong Pan

Last updated by author(s): May 22, 2023

Reporting Summary

Nature Portfolio wishes to improve the reproducibility of the work that we publish. This form provides structure for consistency and transparency in reporting. For further information on Nature Portfolio policies, see our Editorial Policies and the Editorial Policy Checklist.

Statistics

For all statistical analyses, confirm that the following items are present in the figure legend, table legend, main text, or Methods section.

n/a	Confirmed	
☐	☒	The exact sample size (*n*) for each experimental group/condition, given as a discrete number and unit of measurement
☐	☒	A statement on whether measurements were taken from distinct samples or whether the same sample was measured repeatedly
☐	☒	The statistical test(s) used AND whether they are one- or two-sided<br>*Only common tests should be described solely by name; describe more complex techniques in the Methods section.*
☒	☐	A description of all covariates tested
☐	☒	A description of any assumptions or corrections, such as tests of normality and adjustment for multiple comparisons
☐	☒	A full description of the statistical parameters including central tendency (e.g. means) or other basic estimates (e.g. regression coefficient) AND variation (e.g. standard deviation) or associated estimates of uncertainty (e.g. confidence intervals)
☐	☒	For null hypothesis testing, the test statistic (e.g. *F*, *t*, *r*) with confidence intervals, effect sizes, degrees of freedom and *P* value noted<br>*Give P values as exact values whenever suitable.*
☒	☐	For Bayesian analysis, information on the choice of priors and Markov chain Monte Carlo settings
☒	☐	For hierarchical and complex designs, identification of the appropriate level for tests and full reporting of outcomes
☒	☐	Estimates of effect sizes (e.g. Cohen's *d*, Pearson's *r*), indicating how they were calculated

Our web collection on statistics for biologists contains articles on many of the points above.

Software and code

Policy information about availability of computer code

Data collection	Whole genome sequencing of bid1 mutant was performed using illumina platform;<br>Fluorescent Data was collected with Nikon Confocal Imaging Software (Nis-elements viewer v5.21.00);<br>qRT-PCR assay was conducted with CFX96TOUCH(Bio-Rad CFX manager v3.0).<br>Serial section data of nodules was collected by an automated imaging software (AutoSEE):
Data analysis	Statistical analysis was conducted using Graphpad prism (v8.0.2);<br>Pictures of nodule sections for toluidine blue staining were analysed with CaseViewer (v2.4.0.119028);<br>3D reconstruction of serial slices was conducted with Imaris (v9.0.1);<br>Protein 3D structure was predicted by Alphafold Protein Structure Database (https://www.alphafold.ebi.ac.uk/) and merged together with PyMol(v2.5.3);<br>Protein sequence alignment was built with MAFFT (https://mafft.cbrc.jp/alignment/software/) and visualized via ESPript (https://espript.ibcp.fr/ESPript/ESPript/index.php);<br>Phylogenic tree was made using the neighbor-joining method in MEGA 11 (https://megasoftware.net/);<br>Fluorescent intensity of ER structures and measurement of symbiosomes were performed using Image J (v1.53k):<br>SigmaPlot (V14.5) was used to make the histograms in the manuscript.

For manuscripts utilizing custom algorithms or software that are central to the research but not yet described in published literature, software must be made available to editors and reviewers. We strongly encourage code deposition in a community repository (e.g. GitHub). See the Nature Portfolio guidelines for submitting code & software for further information.

Data

Policy information about availability of data

All manuscripts must include a data availability statement. This statement should provide the following information, where applicable:

- Accession codes, unique identifiers, or web links for publicly available datasets
- A description of any restrictions on data availability
- For clinical datasets or third party data, please ensure that the statement adheres to our policy

Source Data generated in this study are provided in the Source Data file, and have also been deposited in the Figshare database under accession code 10.6084/m9.figshare.23077898 [https://doi.org/10.6084/m9.figshare.23077898.v1]. The gene expression data of plant and rhizobial genes mentioned in this study were obtained from Medicago Symbimics Database (https://iant.toulouse.inra.fr/symbimics/) and Medicago Gene Expression Atlas (https://medicago.toulouse.inrae.fr/MtExpress). Additional information is available from the corresponding authors upon request.

Human research participants

Policy information about studies involving human research participants and Sex and Gender in Research.

Reporting on sex and gender	N/A
Population characteristics	N/A
Recruitment	N/A
Ethics oversight	N/A

Note that full information on the approval of the study protocol must also be provided in the manuscript.

Field-specific reporting

Please select the one below that is the best fit for your research. If you are not sure, read the appropriate sections before making your selection.

- [x] Life sciences
- [ ] Behavioural & social sciences
- [ ] Ecological, evolutionary & environmental sciences

For a reference copy of the document with all sections, see nature.com/documents/nr-reporting-summary-flat.pdf

Life sciences study design

All studies must disclose on these points even when the disclosure is negative.

Sample size	Sample size for each experiments are stated in the figure legends and methods of respective experiments. Appropriate sample sizes were chosen based on feasibility of sample collection and common practice in similar studies. Sample sizes were large enough to insure meaningful statistical significance and robust reproducibility.
Data exclusions	No data were excluded.
Replication	Numbers of replications are stated clearly in the figure legends. Experiments were repeated at least three times independently with similar results.
Randomization	Plants were grown in individual pots, plants of different genotypes were allocated randomly in the green house.
Blinding	Experiments were not blinded.

Reporting for specific materials, systems and methods

We require information from authors about some types of materials, experimental systems and methods used in many studies. Here, indicate whether each material, system or method listed is relevant to your study. If you are not sure if a list item applies to your research, read the appropriate section before selecting a response.

Materials & experimental systems

n/a	Involved in the study
[ ]	[x] Antibodies
[x]	[ ] Eukaryotic cell lines
[x]	[ ] Palaeontology and archaeology
[x]	[ ] Animals and other organisms
[x]	[ ] Clinical data
[x]	[ ] Dual use research of concern

Methods

n/a	Involved in the study
[x]	[ ] ChIP-seq
[x]	[ ] Flow cytometry
[x]	[ ] MRI-based neuroimaging

Antibodies

Antibodies used	Anti GFP antibody: GFP Tag(7G9) Mouse mAb(cat.M20004L,Abmart);<br>Second antibody:Goat Anti-Mouse IgG HRP(cat.M21001L,Abmart).
Validation	Validation is based on the datasheet from the manufacturer and the detailed informations are as follows:<br>Anti-GFP antibody(http://www.ab-mart.com.cn/page.aspx?node=%2059%20&id=20971);<br>Goat Anti-Mouse secondary antibody(http://www.ab-mart.com.cn/page.aspx?node=62&id=960).
